# The global trends and clinical progress in influenza co-infection: a visualization and bibliometric analysis (2005–2025)

**DOI:** 10.3389/fmicb.2025.1658752

**Published:** 2025-10-08

**Authors:** Lei Zhang, Shuang Jin, Dabao Ma, Zhiqiang Liu, Jinsheng Ye, Qingquan Liu

**Affiliations:** ^1^Department of Emergency, Yanqing Hospital of Beijing Chinese Medicine Hospital, Beijing, China; ^2^Capital Medical University, Beijing, China; ^3^Department of Surgery, Yanqing Hospital of Beijing Chinese Medicine Hospital, Beijing, China; ^4^Department of Oncology, Yanqing Hospital of Beijing Chinese Medicine Hospital, Beijing, China; ^5^Department of Nephrology, Yanqing Hospital of Beijing Chinese Medicine Hospital, Beijing, China; ^6^Beijing Hospital of Traditional Chinese Medicine, Capital Medical University, Beijing, China; ^7^Laboratory for Clinical Medicine, Capital Medical University, Beijing, China

**Keywords:** influenza, co-infection, COVID-19, children, respiratory syncytial virus, Streptococcus pneumoniae and Staphylococcus aureus

## Abstract

**Objectives:**

Influenza co-infection, characterized by concurrent or sequential infection with influenza and other pathogens, lacks comprehensive quantitative analysis. This study evaluates the status, key hotspots, and clinical advancements in influenza co-infection research from 2005 to 2025 to guide future investigations.

**Methods:**

We analyzed articles from 2005 to 2025 sourced from the Web of Science database using R, VOSviewer, and CiteSpace. Concurrently, we extracted clinical trials from PubMed within the same timeframe to assess advancements in the field.

**Results:**

The study analyzed 3,058 articles, noting a consistent rise in publications on influenza co-infection from 2005 to 2025, with a significant spike between 2020 and 2021. The United States led in publication numbers, followed by China, Germany, the United Kingdom, and France. Among these, the United Kingdom exhibited the highest international collaboration. Key collaborative centers included the Centers for Disease Control and Prevention, Emory University, and St. Jude Children's Research Hospital. “PLOS ONE” and “BMC Infectious Diseases” published the most articles, while “Journal of Virology” and “Journal of Infectious Diseases” were the most cited. Keywords such as “infection”, “virus”, “COVID-19”, “children”, and “respiratory syncytial virus” highlighted research hotspots and emerging trends in influenza co-infection. The study of pathogenic mechanisms and immune interactions in influenza-bacterial co-infection remains crucial. The COVID-19 pandemic has intensified research on the epidemiological shifts and clinical impacts of co-infection. Emphasis has also been placed on the significance of pediatric populations in influenza and respiratory viral co-infections. Clinical trials have mainly targeted preventive strategies for high-risk groups and the effects of influenza vaccination on the respiratory microbiome.

**Conclusion:**

This study comprehensively analyzes the current research landscape and identifies key hotspots in influenza co-infection. The findings offer crucial guidance for future studies in this field.

## 1 Introduction

Influenza represent a persistent and formidable global public health threat, responsible for approximately 1 billion cases annually, including 3–5 million severe cases and 290,000–650,000 deaths worldwide, according to the World Health Organization (World Health Organization, 2025). The virus's substantial genetic variability enables it to trigger recurrent seasonal epidemics and poses unpredictable pandemic risks (Smyk et al., 2022; Morens et al., 2023). While influenza itself causes significant global morbidity and mortality, its impact is often magnified through co-infection with other respiratory pathogens (Bartley et al., 2022; Yan et al., 2023).

Co-infection with influenza occurs when a host is simultaneously or sequentially infected with influenza virus and various pathogens, including *Streptococcus pneumoniae, Staphylococcus aureus* (including methicillin-resistant strains) (Bartley et al., 2022), Haemophilus influenza (Arranz-Herrero et al., 2023), as well as respiratory syncytial virus (RSV) (Haney et al., 2022), SARS-CoV-2 (Yan et al., 2023), and fungi (Feys et al., 2022). It is crucial to recognize that the microbiological diagnosis of co-infection exhibits significant variability depending on the types of pathogens involved. Viral co-infections are generally detected using RT-qPCR or multiplex PCR techniques (Yan et al., 2023), while bacterial co-infections are predominantly verified through quantitative or semi-quantitative sputum and blood cultures (Bartley et al., 2022; Arranz-Herrero et al., 2023). Fungal complications are typically evaluated through a comprehensive methodology that includes culture, antigen or biomarker assays, alongside imaging evidence (Feys et al., 2022). Concurrently, research varies in its application of quantitative thresholds (such as Ct values, viral copy numbers, CFU/mL, antigen indices), sampling intervals, and specimen types (including oropharyngeal/nasopharyngeal swabs, sputum, blood, bronchoalveolar lavage, etc.), and this methodological diversity has a direct impact on detection rates and the clinical interpretation of co-infections (Bartley et al., 2022; Yan et al., 2023; Arranz-Herrero et al., 2023; Feys et al., 2022). Consequently, in the process of defining influenza co-infection, it is essential to take into account the range of pathogens involved as well as the necessary diagnostic criteria and timing considerations. For instance, it is important to classify “concurrent” co-infection as detection occurring within 48 h (Yan et al., 2023), to categorize infections identified during hospital days 1–3 as community-acquired, and to label those identified on days 4–14 as hospital-acquired (Bartley et al., 2022). Cross-study comparability is certainly limited by methodological heterogeneity, but more detailed, pathogen-specific assessments of influenza co-infections have also been made possible by continuous improvements in multiplex diagnostics and improved classification. Concurrently, these developments have resulted in a more precise identification of their clinical detriments.

These co-infections have clinical significance as they are associated with increased severity of influenza, leading to complications such as severe pneumonia, acute respiratory distress syndrome, and multiple organ failure (Yan et al., 2023). Numerous research studies have highlighted that co-infections significantly raise the risks of hospitalization, admission to intensive care units, the requirement for mechanical ventilation, and mortality rates (Bartley et al., 2022; Yan et al., 2023). Some combinations of co-infections have been shown to result in several-fold increases in mortality rates (Arranz-Herrero et al., 2023). Additionally, co-infections often prolong hospital stays, increase healthcare costs, and present challenges in clinical diagnosis due to overlapping symptoms, thereby impeding prompt identification (Bartley et al., 2022; Haney et al., 2022; Chotpitayasunondh et al., 2021). The challenge of influenza co-infection persists, particularly post-COVID-19 pandemic (Chotpitayasunondh et al., 2021). Consequently, an extensive and growing body of research has expanded, reflecting the growing academic interest and progress in this area. Regrettably, the absence of hotspot and frontier analyses in this domain impedes researchers' ability to swiftly and precisely pinpoint future research trajectories.

Bibliometrics serves as a quantitative tool for analyzing scientific literature to development trends, focal points, and frontiers within specific research domains (Lian et al., 2023). A combined quantitative and qualitative bibliometric assessment of influenza co-infection literature elucidates various publication characteristics, such as key contributing countries, journals, authors, and institutions, prominent studies, common keywords, and collaborative networks among countries, institutions, and authors (Song et al., 2019). This analysis offers new researchers an overview of the field's evolution and development trends (Chen et al., 2018). To address this need, the present study utilized R software, VOSviewer, and CiteSpace for a comprehensive analysis of influenza co-infection literature from 2005 to 2025. The study aims to delineate shifts and patterns in research focal points within this domain and pinpoint potential areas for future investigation. Understanding the current landscape and prospects of influenza co-infection is crucial for its sustainable advancement.

## 2 Materials and methods

### 2.1 Data collection

**Figure 1:**
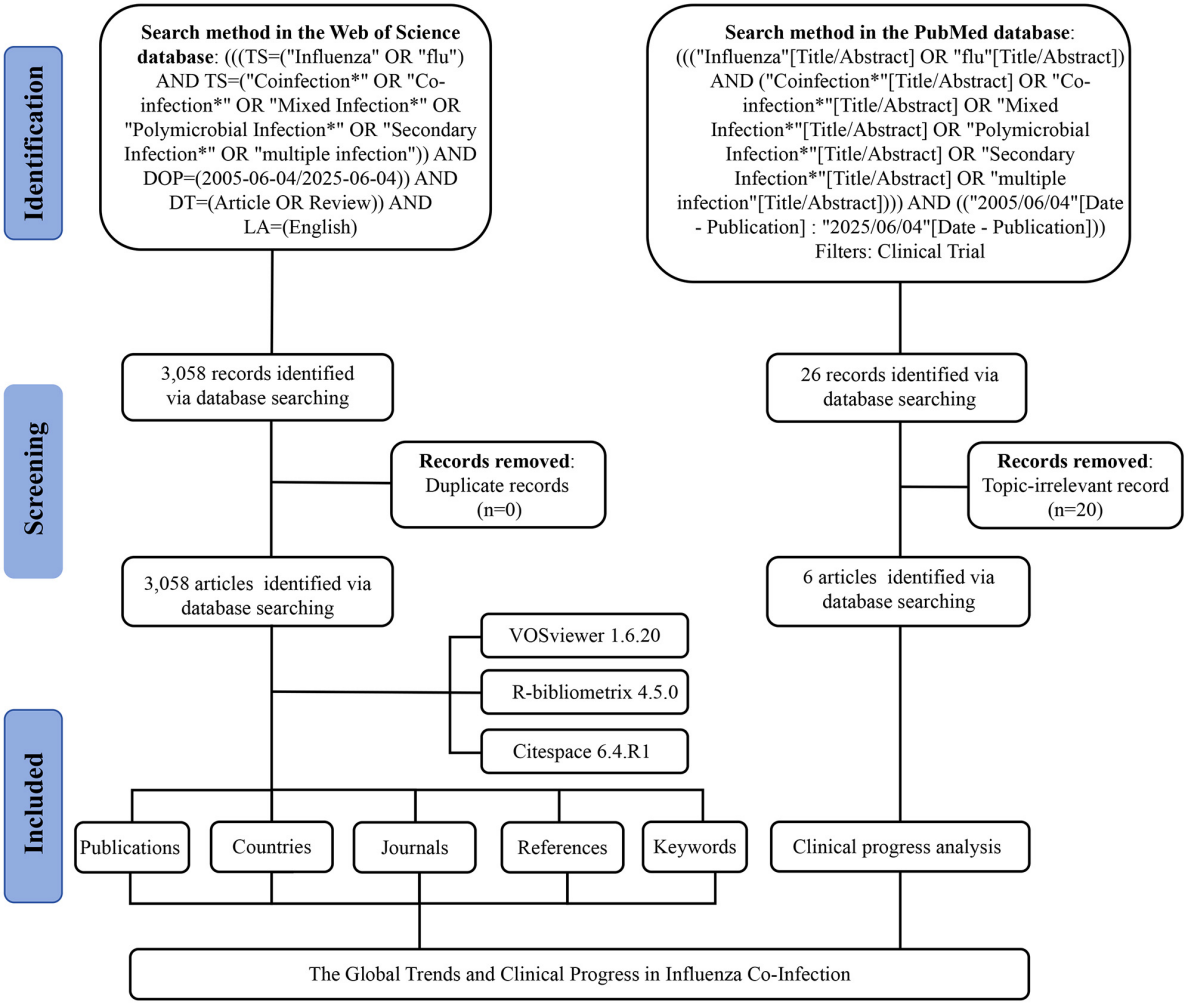
A flow chart of publication retrieval.

The data for this study were retrieved from the Web of Science (Capital Medical University Edition) and PubMed databases on June 4, 2025. Details of the search strategy are provided in Figure 1. Articles and review articles were included, without distinguishing pathogen types, sample collection time, and other such factors during the inclusion or analysis phases. Duplicate records were excluded. The remaining articles were saved in plain text format, and their references were exported as full records. Clinical trial results were additionally exported in PubMed format. Subsequently, two researchers undertook a manual screening of clinical trial entries, meticulously examining titles, abstracts, and keywords to systematically eliminate studies that were not pertinent to influenza or co-infection, thus maintaining a high level of topical relevance. Discrepancies among reviewers were addressed through discussion, and, when required, adjudicated by a third researcher.

### 2.2 Data analysis

Origin 2018 was used to analyze annual publication trends. Further data visualization and scientific knowledge mapping were performed using R software (version 4.5.0) (http://www.bibliometrix.org) (Aria and Cuccurullo, 2017), VOSviewer (version 1.6.20) (van Eck and Waltman, 2010), and CiteSpace (version 6.4.R1) (Chen, 2006), in combination with the bibliometrix package. To maintain accuracy and reliability, two independent authors conducted all data extraction and analytical procedures separately.

VOSviewer was employed to visualize co-authorship networks among countries and institutions, conduct co-citation analysis of sources, and explore keyword co-occurrence. For co-authorship analysis, the minimum number of documents was set at five for each country and 15 for each organization. In the co-citation analysis of sources, only those with at least 104 citations were included. For keyword co-occurrence, the minimum occurrence threshold was set at twenty-five, excluding general terms such as “Influenza” and “Co-Infection.” The analysis was conducted using Journal Impact Factors (IFs) from the 2024 edition of the Journal Citation Reports (JCR).

## 3 Results

### 3.1 General landscapes of global publications

**Figure 2:**
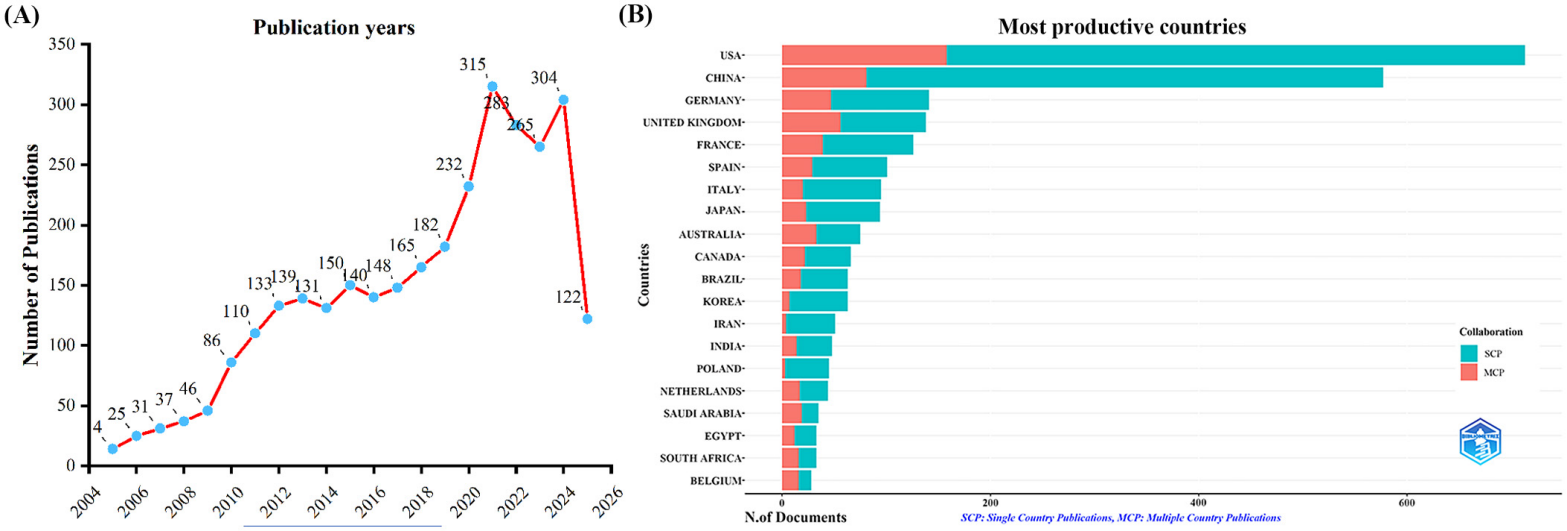
Annual publication trends in influenza co-infection, 2005–2025. **(A)** Yearly publication trends. **(B)** Country and collaboration distribution of corresponding authors.

A total of 3,058 publications were retrieved from the Web of Science Core Collection (WoSCC) database, including 2,729 articles and 329 reviews. We constructed a line chart to illustrate the annual trend in the number of publications on influenza co-infection from 2005 to 2025 (Figure 2A). Based on the yearly growth rate of publications, the study period was divided into the following four phases: the first phase (2005–2009) was characterized by slow growth; the second phase (2010–2019) exhibited steady growth; the third phase (2020–2021) demonstrated rapid increase; and the fourth phase (2021–2025) represented a period of high-level fluctuation, primarily influenced by the 2009 H1N1 influenza pandemic and the emergence of the COVID-19 pandemic in 2019, impacting publication trends during these periods.

**Figure 3:**
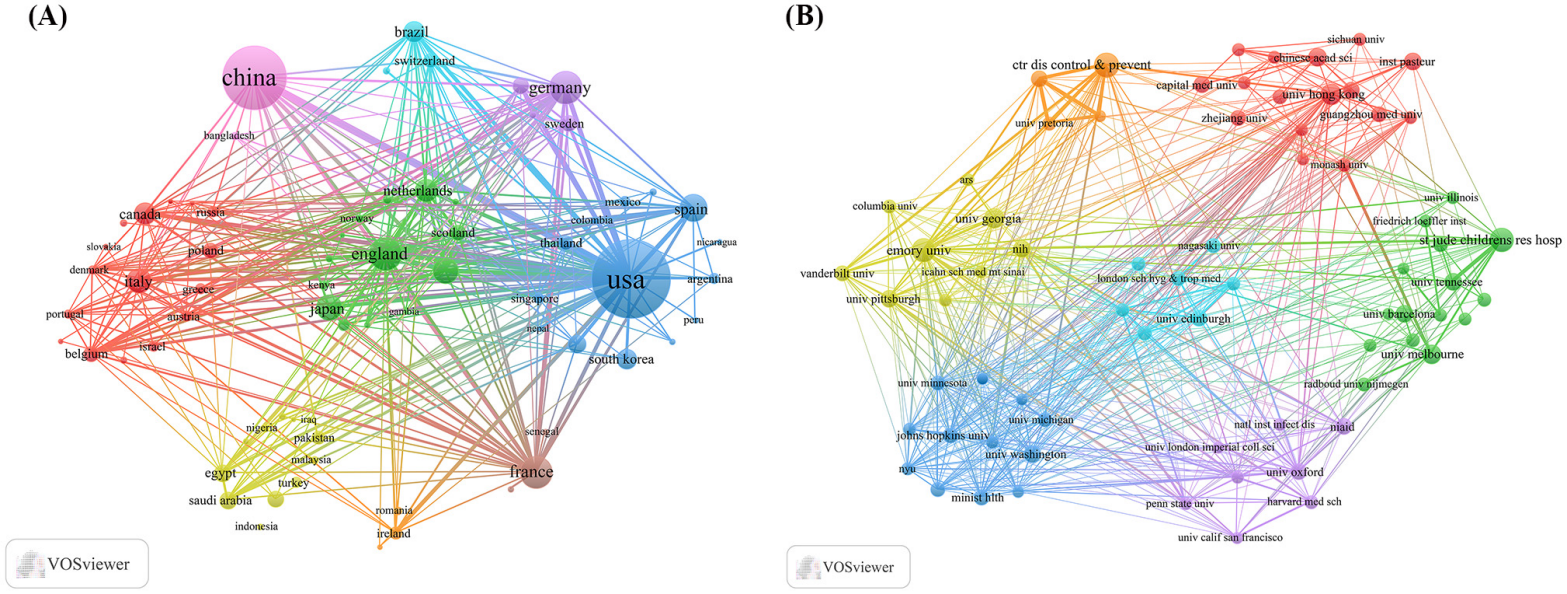
Map of countries/regions and institutions in influenza co-infection, 2005–2025. **(A)** Country collaboration map. **(B)** Institutional collaboration map.

By examining the countries of corresponding authors, it was observed that the USA (*n* = 713) was the leading contributor in this field, followed by China (*n* = 577), Germany (*n* = 141), the United Kingdom (*n* = 138), and France (*n* = 126). Among the top five most productive countries, the USA ranked first both in total articles and single-country publications (SCP, *n* = 555), with a multi-country publication (MCP) rate of 22.2%. China held the second position in both total publications and SCPs (*n* = 496); however, only 14.0% of its articles were MCPs, which was notably lower than the other leading countries (Figure 2B, Table 1). In contrast, while the United Kingdom exhibited a lower total number of publications, the percentage of MCPs attained 40.6%, representing the highest figure among the leading five nations. Ireland, Belgium, and Saudi Arabia, despite producing a lower volume of publications overall, exhibited notable MCP rates of 75.0%, 57.1%, and 54.3%, respectively. Furthermore, Figure 3A reveals extensive international collaboration among nations within this domain. The collaboration analysis also identified the Centers for Disease Control and Prevention (CDC, *n* = 66), Emory University (*n* = 62), and St. Jude Children's Research Hospital (*n* = 61) as prominent collaboration centers (Figure 3B, Table 2).

**Table 1:** Most relevant countries of corresponding authors in influenza co-infection research.

**Country**	**Articles**	**SCP**	**MCP**	**Freq**	**MCP_Ratio**
USA	713	555	158	23.3	22.2
China	577	496	81	18.9	14
Germany	141	94	47	4.6	33.3
United Kingdom	138	82	56	4.5	40.6
France	126	87	39	4.1	31
Spain	101	72	29	3.3	28.7
Italy	95	75	20	3.1	21.1
Japan	94	71	23	3.1	24.5
Australia	75	42	33	2.5	44
Canada	66	44	22	2.2	33.3
Brazil	63	45	18	2.1	28.6
Korea	63	56	7	2.1	11.1
Iran	51	47	4	1.7	7.8
India	48	34	14	1.6	29.2
Poland	45	42	3	1.5	6.7
Netherlands	44	27	17	1.4	38.6
Saudi Arabia	35	16	19	1.1	54.3
Egypt	33	21	12	1.1	36.4
South Africa	33	17	16	1.1	48.5
Belgium	28	12	16	0.9	57.1
Thailand	25	19	6	0.8	24
Sweden	23	11	12	0.8	52.2
Switzerland	21	12	9	0.7	42.9
Turkey	21	20	1	0.7	4.8
Ireland	20	5	15	0.7	75

MCP, Multiple country publication; SCP, Single country publication.

**Table 2:** Most relevant author affiliations in influenza co-infection research.

**Affiliation**	**Articles (*n*)**
Centers for Disease Control and Prevention	66
Emory University	62
St. Jude Children's Research Hospital	61
The University of Hong Kong	48
University of Georgia	43
The University of Melbourne	40
Chinese Academy of Sciences	34
University of Tennessee	32
Institut Pasteur	31
University of Pittsburgh	31
National Institutes of Health	30
University of Barcelona	30
University of Washington	30
National Institute of Allergy and Infectious Diseases	29
University of Oxford	29
University of Edinburgh	28
Capital Medical University	27
Fudan University	27
University of the Witwatersrand	27
Guangzhou Medical University	26

### 3.2 Journals and co-cited journals

**Figure 4:**
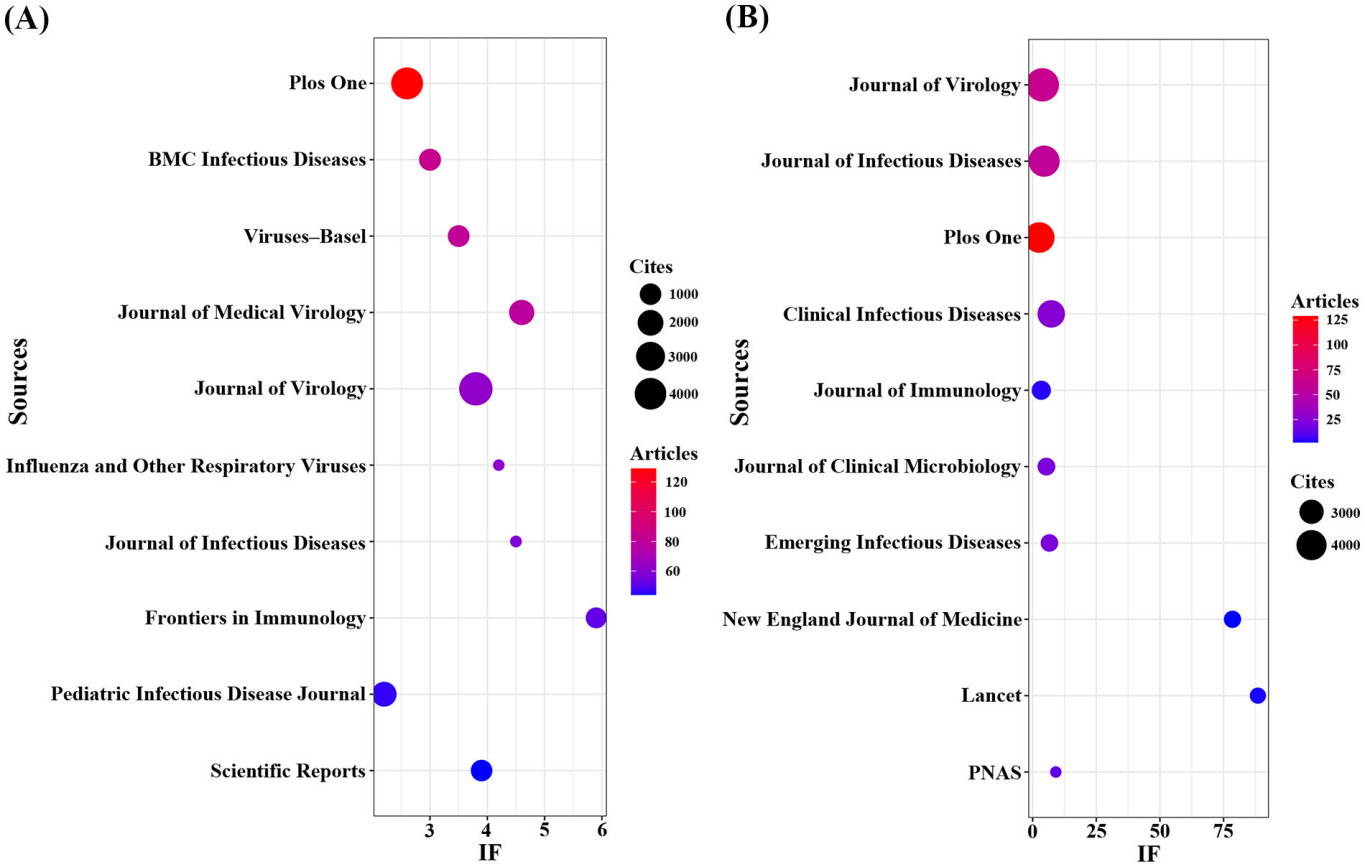
Journals with the most publications/citations. **(A)** Journals with the most publications **(B)** Journals with the most citations.

**Figure 5:**
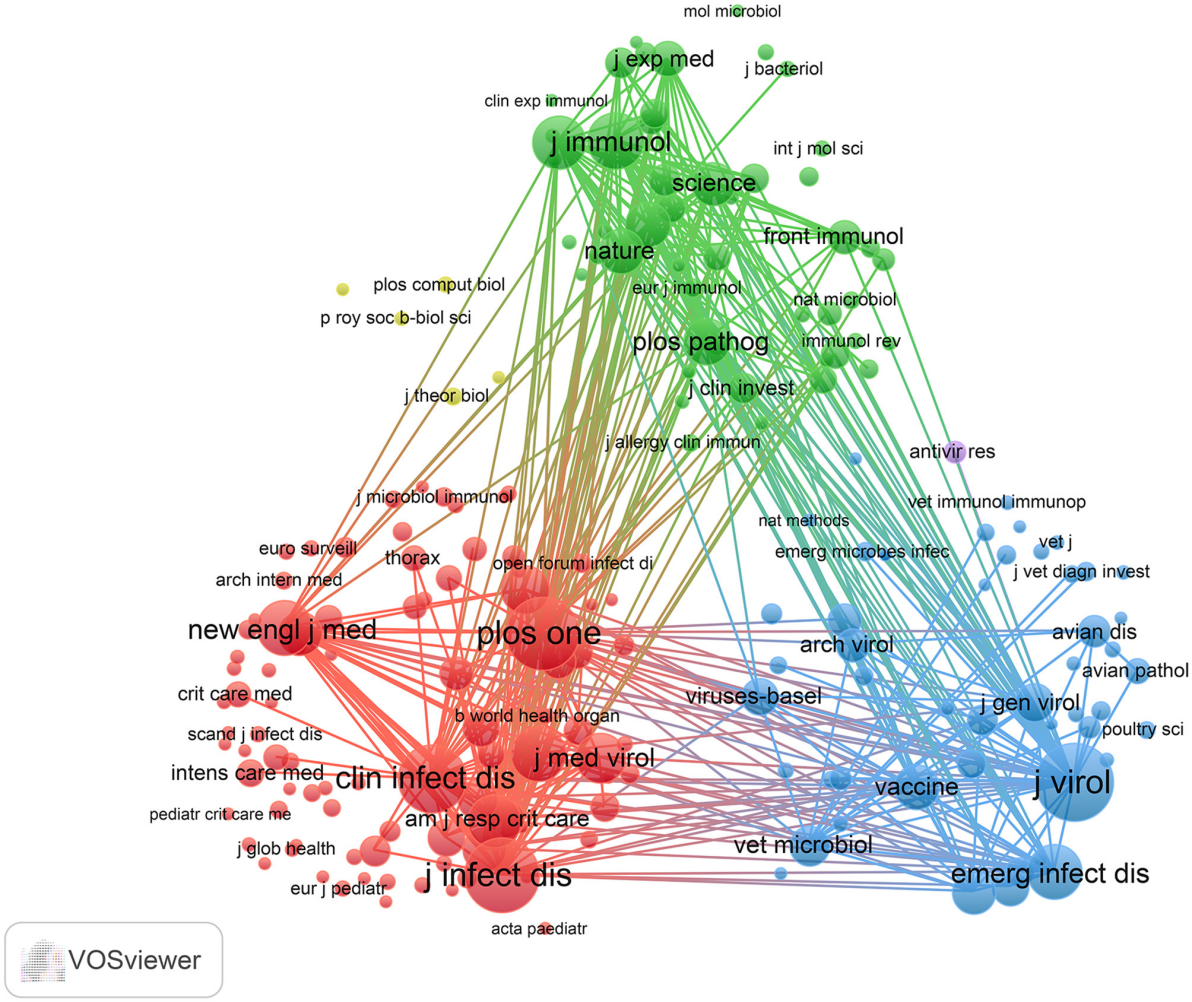
Co-citation analysis of journals in influenza co-infection.

Using R software (version 4.5.0) with the Bibliometrix and ggplot2 packages, together with VOSviewer (version 1.6.20) for co-citation journal analysis, a total of 3,058 articles were identified across 681 academic journals (Annex 1). As shown in Table 3 and Figure 4A, “Plos One” published the highest number of articles (*n* = 129, IF = 2.6), followed by “BMC Infectious Diseases” (*n* = 85, IF = 3.0), “Viruses-Basel” (*n* = 81, IF = 3.5), “Journal of Medical Virology” (*n* = 79, IF = 4.6), and “Journal of Virology” (*n* = 63, IF = 3.8). Furthermore, Table 4 and Figure 4B present the most frequently cited journals, with “Journal of Virology” (*n* = 4,719, IF = 3.8) leading, followed by “Journal of Infectious Diseases” (*n* = 4,231, IF = 4.5), “Plos One” (*n* = 4,104, IF = 2.6), “Clinical Infectious Diseases” (*n* = 3,503, IF = 7.3), and “Journal of Immunology” (*n* = 2,434, IF = 3.4). The co-citation journal map indicates that “Journal of Virology”, “Journal of Infectious Diseases”, and “Plos One” serve as key collaborative hubs within the field (Figure 5). These results suggest that “Journal of Virology” and “Plos One” may represent influential journals in influenza co-infection research. Additionally, the data reveal a notable scarcity of publications in prestigious journals within this domain, emphasizing the necessity to improve both the depth and quality of associated research.

**Table 3:** Top 10 most published journals.

**Journal**	**Documents**	**IF(2024)**	**Cites**
PLoS ONE	129	2.6	4,104
BMC Infectious Diseases	85	3.0	1,083
Viruses-Basel	81	3.5	1,052
Journal of Medical Virology	79	4.6	1,883
Journal of Virology	63	3.8	4,719
Influenza and Other Respiratory Viruses	62	4.2	1,431
Journal of Infectious Diseases	58	4.5	4,231
Frontiers in Immunology	52	5.9	875
Pediatric Infectious Disease Journal	46	2.2	1,770
Scientific Reports	44	3.9	1,003

**Table 4:** Top 10 most cited journals.

**Journal**	**Cites**	**If(2024)**	**Document**
Journal of Virology	4,719	3.8	63
Journal of Infectious Diseases	4,231	4.5	58
Plos One	4,104	2.6	129
Clinical Infectious Diseases	3,503	7.3	25
Journal of Immunology	2,434	3.4	29
Journal of Clinical Microbiology	2,328	5.4	21
Emerging Infectious Diseases	2,309	6.6	21
New England Journal of Medicine	2,292	78.5	2
Lancet	2,228	88.5	3
PNAS	2,106	9.1	13

### 3.3 Analysis of cited-references

We utilized the bibliometrix package in R software to identify the top 25 most cited publications in the domain of influenza co-infection (Table 5). Each of these references has been cited at least 303 times and they are distributed across 20 different journals, indicating that substantial breakthroughs are still lacking in this area. Notably, there is no single dominant journal among the top 25 cited works. The three most cited articles are “Co-infections in patients with COVID-19: A systematic review and meta-analysis,” “Pneumonia and respiratory failure from swine-origin influenza A (H1N1) in Mexico,” and “Bacterial co-infection and secondary infection in patients with COVID-19: a living rapid review and meta-analysis.” Upon further analysis, we observed that these publications mainly provide general overviews concerning infection types, epidemiology, and clinical characteristics in the context of influenza co-infection.

**Table 5:** Top 20 most cited references on influenza co-infection.

**Paper**	**DOI**	**Total citations**	**TC per year**
Lansbury L, 2020, J Infection	10.1016/j.jinf.2020.05.046	1,047	174.50
Perez-Padilla R, 2009, New Engl J Med	10.1056/NEJMoa0904252	1,030	60.59
Langford BJ, 2020, Clin Microbiol Infec	10.1016/j.cmi.2020.07.016	1,015	169.17
Ruuskanen O, 2011, Lancet	10.1016/S0140-6736(10)61459-6	864	57.60
Heffernan JM, 2005, J R Soc Interface	10.1098/rsif.2005.0042	787	37.48
Chen Y, 2013, Lancet	10.1016/S0140-6736(13)60903-4	680	52.31
Schauwvlieghe Afad, 2018, Lancet Resp Med	10.1016/S2213-2600(18)30274-1	656	82.00
Garcia-Vidal C, 2021, Clin Microbiol Infec	10.1016/j.cmi.2020.07.041	633	126.60
Bente DA, 2013, Antivir Res	10.1016/j.antiviral.2013.07.006	622	47.85
Chen HY, 2014, Lancet	10.1016/S0140-6736(14)60111-2	525	43.75
Chen TM, 2020, Infect Dis Poverty	10.1186/s40249-020-00640-3	512	85.33
Bhat N, 2005, New Engl J Med	10.1056/NEJMoa051721	495	23.57
Hughes S, 2020, Clin Microbiol Infec	10.1016/j.cmi.2020.06.025	447	74.50
Klein EY, 2016, Influenza Other Resp	10.1111/irv.12398	369	36.90
Morris DE, 2017, Front Microbiol	10.3389/fmicb.2017.01041	359	39.89
To KKW, 2010, Clin Infect Dis	10.1086/650581	359	22.44
Musuuza JS, 2021, PLoS ONE	10.1371/journal.pone.0251170	342	68.40
Shieh WJ, 2010, Am J Pathol	10.2353/ajpath.2010.100115	332	20.75
Plourde AR, 2016, Emerg Infect Dis	10.3201/eid2207.151990	322	32.20
Lai CC, 2020, J Microbiol Immunol	10.1016/j.jmii.2020.05.013	320	53.33
Nickbakhsh S, 2019, P Natl Acad Sci USA	10.1073/pnas.1911083116	320	45.71
Kalil AC, 2019, Crit Care	10.1186/s13054-019-2539-x	317	45.29
Chertow DS, 2013, JAMA-J Am Med Assoc	10.1001/jama.2012.194139	315	24.23
Ding Q, 2020, J Med Virol	10.1002/jmv.25781	306	51.00
Jennings LC, 2008, Thorax	10.1136/thx.2006.075077	304	16.89

**Figure 6:**
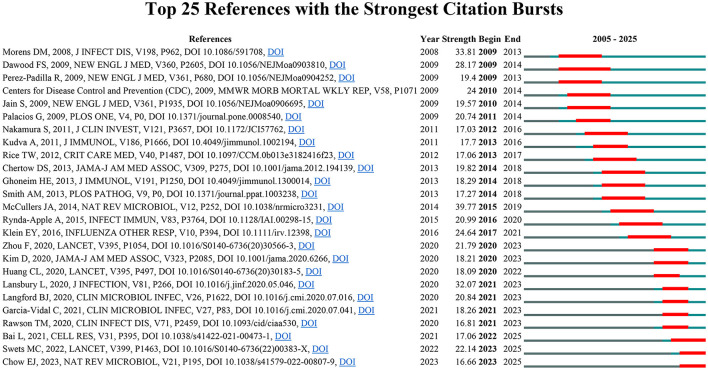
Top 25 most cited references in influenza co-infection.

To systematically evaluate the major research themes and emerging issues in the field of influenza co-infection, we employed CiteSpace software to identify 435 references with marked citation bursts, according to predetermined criteria (top 25; status count: 2; minimum duration: 2). A representative selection of 25 is depicted in Figure 6. The full list of these references, together with their corresponding DOIs, is provided in Annex 2. Notably, the three references with the highest citation burst strengths were: “Bacterial complications during pandemic influenza infection” (strength: 39.77), “Emergence of a novel swine-origin influenza A (H1N1) virus in humans” (strength: 33.81), and “Pneumonia and respiratory failure from swine-origin influenza A (H1N1) in Mexico” (strength: 32.07). In addition, the most recent citation bursts were associated with the following articles: (1) “Coinfection with influenza A virus enhances SARS-CoV-2 infectivity,” (2) “SARS-CoV-2 co-infection with influenza viruses, RSV, or adenoviruses,” and (3) “The effects of the COVID-19 pandemic on community respiratory virus activity”.

In summary, based on the citation burst analysis, we have identified three principal research hotspots in the field of influenza co-infection: (1) The epidemiological and clinical characteristics of influenza co-infection; (2) The pathological and immunological interplay between influenza viruses and co-infecting pathogens; (3) The interaction between COVID-19 and influenza.

### 3.4 Keyword clusters and evolution

Keyword cluster analysis is an effective tool for identifying research hotspots and developmental trends within academic fields. In this study, we applied VOSviewer software to extract 7,990 keywords from the current literature. Table 6 provides a comprehensive overview of keyword frequency, indicating that 20 terms appeared more than 151 times. Notably, “infection” ranked first with 694 occurrences, followed by “virus” (*n* = 636), “covid-19” (*n* = 495), “children” (*n* = 428), “respiratory syncytial virus” (*n* = 420), “pneumonia” (*n* = 332), “influenza a virus” (*n* = 323), and “*streptococcus pneumoniae*” (*n* = 279).

**Table 6:** Top 20 keywords related to influenza co-infection.

**Rank**	**Words**	**Occurrences**
1	Infection	694
2	Virus	636
3	COVID-19	495
4	Children	428
5	Respiratory syncytial virus	420
6	Pneumonia	332
7	Influenza A virus	323
8	*Streptococcus pneumoniae*	279
9	Epidemiology	278
10	Disease	255
11	Respiratory tract infection	231
12	Community-acquired pneumonia	213
13	Respiratory virus	197
14	Pandemic influenza	195
15	United-States	176
16	Viral infection	167
17	Mortality	166
18	Polymerase-chain-reaction	166
19	Transmission	161
20	Diagnosis	151

**Figure 7:**
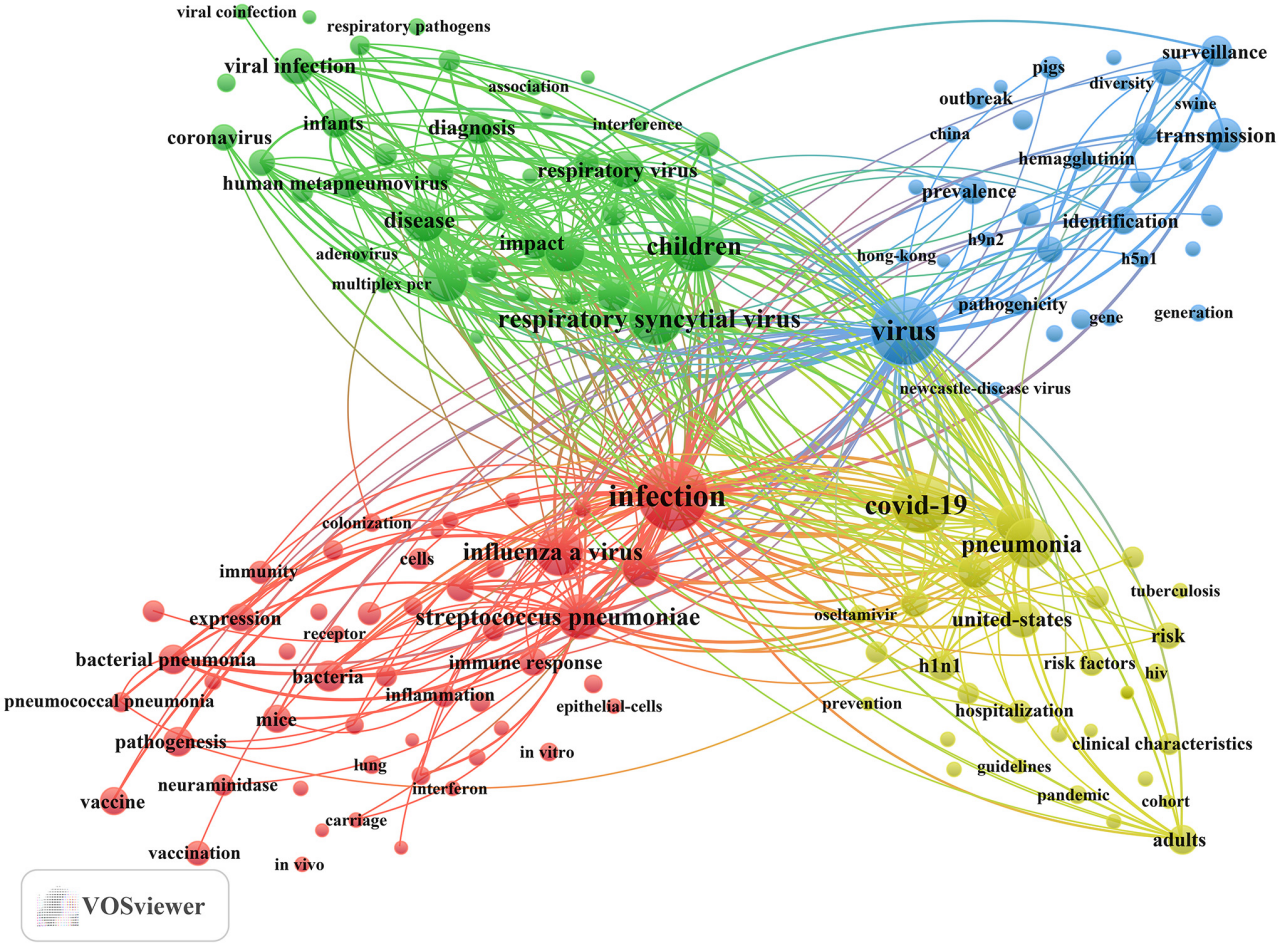
Keyword co-occurrence map for influenza co-infection publications.

In addition, we identified 195 keywords with a minimum occurrence of 25 times and constructed a keyword cluster map using these terms (Figure 7). The map presents four distinct clusters, each represented by a different color. Utilizing cluster analysis, we identified four major thematic clusters that reflect the current research frontiers in influenza co-infection. Cluster 1 (red dots) focuses on the fundamental mechanisms underlying co-infections between influenza viruses and bacteria, particularly *Streptococcus pneumoniae* and *Staphylococcus aureus*. Key terms include infection, influenza A virus, *Streptococcus pneumoniae*, pandemic influenza, and bacteria. Cluster 2 (green dots) centers on pediatric populations, exploring the epidemiological features, clinical diagnostic methods, and disease burden associated with co-infections caused by influenza and other respiratory viruses, such as RSV. This cluster underscores the susceptibility of children to multiple viral co-infections and the significance of this group as a priority in clinical research. Relevant terms include children, RSV, epidemiology, disease, and respiratory tract infection. Cluster 3 (blue dots) highlights research on the evolutionary trajectories, cross-species transmission, and global surveillance of influenza, especially zoonotic viruses, such as avian influenza. Key terms in this cluster include virus, transmission, surveillance, evolution, and identification. Cluster 4 (yellow dots) captures the changing landscape of multi-pathogen co-infection during the COVID-19 pandemic, with a particular focus on variations in the incidence, severity, and mortality of community-acquired pneumonia involving influenza and other respiratory pathogens. Core keywords encompass COVID-19, pneumonia, community-acquired pneumonia, United States, and mortality. The full list of keywords corresponding to these four clusters is provided in Annex 3.

In order to connect these indicators with disease burden, patient characteristics, and decision-making in clinical care and epidemiology, our analysis of keywords underscores the following significant areas of research in influenza and co-infection: (1) Priority populations encompass infants, children, hospitalized patients, and those who are critically ill (e.g., “children,” “young children,” “infants,” “hospitalized children,” “critically-ill patients”). Individuals with compromised immune systems and those with comorbid conditions represent significant populations (e.g., “HIV,” “tuberculosis”). Outcome metrics concentrate on the aspects of hospitalization and mortality. (2) Co-infection constellations focus on influenza A and its various subtypes, such as “H1N1,” “H3N2,” and “H5N1.” High-frequency patterns encompass the following combinations: “influenza + *streptococcus pneumoniae*,” “influenza + *staphylococcus aureus*,” “influenza + RSV,” “influenza + human metapneumovirus,” “influenza + rhinovirus,” “influenza + coronavirus (including SARS-CoV-2),” “influenza + human bocavirus,” “influenza + adenovirus,” “influenza + parainfluenza virus,” and “influenza + newcastle-disease virus.” (3) Geographic hotspots suggest that China and the United States are prominent regions for high-output research and serve as critical settings for epidemiological studies and priority populations, which aligns with national analyses. (4) Populations with significant exposure associated with poultry and livestock represent a concurrent area of interest. Risk is concentrated in occupational and environmental contexts associated with poultry, swine, pigs, chickens, and wild birds, which is indicative of their close relationship with outbreaks of highly pathogenic avian influenza and instances of cross-species transmission. (5) Interventions and prevention strategies encompass vaccination (e.g., “influenza vaccination,” “conjugate vaccine”), antiviral and antimicrobial management (e.g., “oseltamivir,” “procalcitonin,” “c-reactive protein”), multiplex molecular diagnostics (e.g., “real-time PCR,” “multiplex PCR”), as well as public health surveillance and infection control measures (e.g., “surveillance,” “seasonality,” “guidelines”). The integration of vaccination strategies alongside the focused management of high-risk populations is identified as a critical area for both clinical and policy intervention (Annex 3).

**Figure 8:**
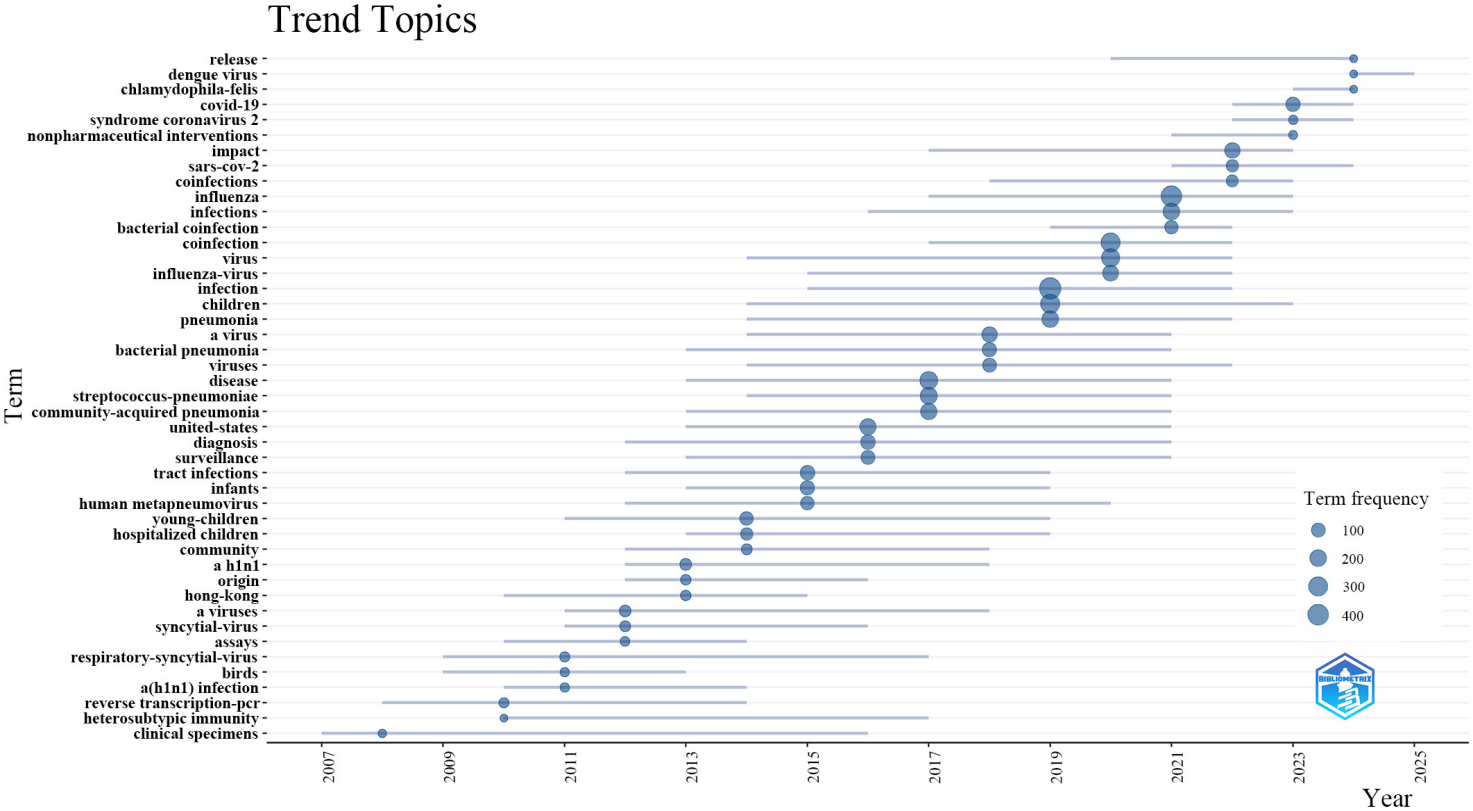
Trending topics in influenza co-infection.

Additionally, to elucidate temporal shifts and forecast trends in influenza co-infection research, we utilized the bibliometrix toolkit in R to create a visual trend topic chart (Figure 8). From 2007 to 2013, research primarily focused on identifying and monitoring respiratory pathogens, with an emphasis on assay development, viral origins, and clinical specimen analysis. Foundational studies during this period concentrated on influenza virus subtypes, such as H1N1, and zoonotic reservoirs, particularly birds, setting the stage for future research. Between 2013 and 2017, the focus shifted toward the epidemiology and clinical impact of pediatric respiratory infections, including RSV, human metapneumovirus, and co-infection patterns in infants and young children. During this phase, terms related to diagnostic innovations and community-acquired pneumonia emerged. From 2017, studies increasingly focused on bacterial co-infections, antibacterial resistance, and the immunological interactions between influenza viruses and bacterial pathogens like *Streptococcus pneumoniae*. Since 2022, interest has surged in COVID-19, SARS-CoV-2, and the clinical impact of viral co-infections, including nonpharmaceutical interventions and the global effects of pandemics. Our analysis suggests that research on influenza co-infection will likely intensify, concentrating on co-pathogenesis mechanisms, clinical risk assessment, and interactions between emerging respiratory viruses and bacterial agents, with particular attention to vaccine development and population-level management.

### 3.5 Clinical progress analysis

Six clinical trials were identified from the PubMed database (Annex 4). The following work presents a concise overview of the principal arguments: (1) The examination of host genetic factors indicated that low-expression variants of the mannose-binding lectin 2 gene did not demonstrate a significant correlation with heightened overall susceptibility or severity of critical influenza in pediatric populations. In the subgroup of influenza associated with methicillin-resistant staphylococcus aureus co-infection, an increased frequency of the B allele among carriers was noted. (2) In pediatric patients diagnosed with hematologic malignancies and receiving chemotherapy, community-acquired influenza and parainfluenza viruses have been recognized as prevalent pathogens associated with lower respiratory tract infections, which primarily demonstrated self-limiting trajectories. Under conditions of immunosuppression, the association of viral–bacterial co-infection with rapid and severe disease progression was observed. (3) The implementation of N95 respirators among healthcare workers has been shown to markedly decrease the occurrence of upper respiratory bacterial colonization and viral–bacterial co-infection, achieving an efficacy rate of 66% in comparison to medical masks. (4) In Gambian children aged 24–59 months, the administration of live attenuated influenza vaccine (LAIV) vaccination resulted in only mild and transient alterations in the nasopharyngeal microbiota. The observed shifts were shaped by environmental factors. Moreover, the composition of baseline microbiota exhibited a significant correlation with T-cell immune responses following vaccination. (5) In the same research team, the administration of LAIV led to a temporary elevation in the density of nasopharyngeal Streptococcus pneumoniae (1.3–1.9-fold, significant on D7 and D21), especially in children exhibiting asymptomatic respiratory viral co-infections or increased vaccine virus shedding. No clinically significant adverse events were documented. (6) A further investigation revealed that, although LAIV resulted in a lower incidence of symptoms following bacterial exposure when compared to the inactivated influenza vaccine, it was linked to markedly elevated rates and density of pneumococcal colonization. These trials primarily investigate two key areas in this field: (1) Unique host types and strategies for preventing influenza co-infection; (2) The influence of influenza vaccines on the microbiota of the respiratory tract and their correlation with co-infection.

## 4 Discussion

### 4.1 General information

The present study conducted a comprehensive bibliometric and visual analysis of 3,058 publications on influenza co-infection from 2005 to 2025. The results reveal a distinct upward trend in the volume of literature over this period. Specifically, the study period can be divided into four phases based on the annual publication growth rates: (1) slow growth from 2005 to 2009, (2) steady increases from 2010 to 2019, (3) rapid expansion from 2020 to 2021, (4) sustained high-level fluctuations from 2021 to 2025. These publication trends closely mirror two major public health events: the 2009 H1N1 influenza pandemic (Fineberg, 2014) and the onset of the COVID-19 pandemic in 2019 (Yan et al., 2023; Chotpitayasunondh et al., 2021). It is important to note that the data for 2025 are incomplete, as the search was conducted on June 4, 2025.

The United States is the global leader in published research on influenza co-infection, with 713 articles, demonstrating the country's substantial research interest and contributions in this field. China ranks second with 577 publications, while Germany, the United Kingdom, and France have also made significant advances. Among the top 20 most prolific institutions, the U.S.-based CDC, Emory University, and St. Jude Children's Research Hospital lead with 66, 58, and 48 publications, respectively, underscoring the U.S. dominance. However, institutions from China, Australia, France, Spain, and the United Kingdom are also well-represented, reflecting the global attention and collaborative efforts in this research domain. Additionally, the CDC has historically taken the initiative or partnered in the establishment of comprehensive nationwide programs for influenza sentinel surveillance, hospitalization surveillance, pathogen spectrum surveillance, and co-infection surveillance. The accumulation of high-quality, reusable large-scale clinical and molecular epidemiological datasets has established a robust data foundation for multicenter studies and expedited publication processes, resulting in a significant increase in research output. From an

institutional ecology perspective, a notable hub-aggregation effect is apparent: the CDC, Emory University, and St. Jude Children's Research Hospital have participated in ongoing efforts in the fields of influenza, virology, pediatric infections, and vaccinology, thereby creating a consistent integrated production mechanism characterized by “methods–samples–clinical questions.” They integrate significant centrality with bridging functions, thus enhancing the effectiveness of collaboration across institutions and nations. In alignment with the analysis conducted, pediatric populations represent a significant area of research focus. This is in strong agreement with the capabilities of St. Jude Children's Research Hospital, which is well-known for its research in pediatrics and severe infections, and is ranked among the top three institutions based on publication count. This indicates a favorable relationship between the areas of collaboration hubs and thematic hotspots.

The extant literature on influenza co-infection is extensive, spanning 3,058 articles published across 681 academic journals. The journals with the highest publication output in this domain are PLOS ONE, BMC Infectious Diseases, and Viruses-Basel, underscoring their substantial contributions to the development of this field. Furthermore, Journal of Virology and PLOS ONE emerge as the most frequently cited journals, serving as central hubs of journal collaboration and thus highlighting their representative and influential role within this research area.

### 4.2 Hotspots and development trends

As mentioned above, by conducting a comprehensive analysis of literature clustering, keyword frequency, keyword co-occurrence, and research topic evolution, we identified emerging research hotspots in influenza co-infection. The findings emphasize three primary areas: First, the pathogenic mechanisms and immune interactions in influenza–bacterial coinfections remain a core focus, highlighting how synergistic effects worsen clinical outcomes. Second, the COVID-19 pandemic has caused epidemiological shifts and added complexity to the clinical burden of influenza coinfections, emphasizing the need for updated surveillance and management. Third, the epidemiology and precision management of influenza–respiratory virus coinfection in children require special attention due to their increased susceptibility and unique clinical needs. It should be noted that the research findings are not prescriptive inferences but rather the result of a systematic examination of the body of existing knowledge.

#### 4.2.1 Pathogenic mechanisms and immune interactions in influenza-bacterial coinfection

According to bibliometric analysis, the topic of “pathogenic mechanisms and immune interactions” has been identified as a significant research hotspot within the domain of influenza coinfection. The interaction between influenza viruses and bacterial pathogens is crucial in infection biology. Influenza infection increases susceptibility to secondary bacterial infections, worsening disease severity and mortality (Aguilera and Lenz, 2020; Morris et al., 2017). *Streptococcus pneumoniae* and *Staphylococcus aureus* are the most common secondary bacterial pathogens, while *Haemophilus influenzae, Klebsiella pneumoniae*, and *Moraxella catarrhalis* also play important roles among Gram-negative bacteria (Morris et al., 2017; Sender et al., 2021; Huo et al., 2025).

Numerous aspects reveal the synergistic effects of influenza viruses and bacteria. Firstly, influenza viruses can promote the adhesion of bacteria to respiratory epithelial cells during respiratory tract infections, resulting in a higher bacterial load in the tissues (Rowe et al., 2019). Secondly, influenza-induced damage to the respiratory epithelium impairs mucociliary clearance and exposes additional bacterial adhesion sites, greatly increasing bacterial attachment and invasion (Sender et al., 2021; Huo et al., 2025; Oliva and Terrier, 2021). Thirdly, type I and type III interferons induced by influenza viruses delay epithelial repair by inhibiting cell proliferation and differentiation and promoting apoptosis, increasing the risk of secondary bacterial infections and worsening disease severity (Oliva and Terrier, 2021; Major et al., 2020). In addition, the dysregulation of the host immune response also plays a crucial role (Aguilera and Lenz, 2020). While type I interferons are vital for antiviral defense, their excessive activation impairs antibacterial responses by reducing neutrophil recruitment and disrupting alveolar macrophages, promoting bacterial survival and spread. Co-infection induces a synergistic cytokine storm—with elevated IL-6, IL-1β, TNF-α, and MCP-1—further increasing susceptibility to secondary bacterial infections and worsening tissue damage and disease severity (Sender et al., 2021; Oliva and Terrier, 2021; Zangari et al., 2021). At the same time, the adaptive immune response, involving CD8+/CD4+ T cells and antibodies, is essential for clearing secondary bacterial infections, but factors like weakened Th17 responses, inhibited T-cell function, and reduced antibacterial cytokines can compromise this protection and raise the risk of secondary infections (Sender et al., 2021; Oliva and Terrier, 2021). Furthermore, following co-infection with influenza virus and *Staphylococcus aureus*, mitochondrial autophagy in lung epithelial cells inhibits apoptosis, promoting viral and bacterial proliferation, worsening inflammation and pneumonia, and ultimately lowering survival rates (Huo et al., 2025). Additionally, influenza and bacterial neuraminidases synergistically cleave sialic acid on host cells, enhancing bacterial adhesion and colonization in the respiratory tract and worsening the pathological damage of secondary infections and co-infections (Alshammari et al., 2025). Ultimately, secondary bacterial pathogens exploit virus-induced immunosuppression and tissue damage to increase their proliferation and pathogenicity. Influenza-altered alveolar macrophages have reduced phagocytic and bactericidal functions, leading to higher bacterial loads and worse clinical outcomes (Lee et al., 2018; Deinhardt-Emmer et al., 2020).

Future research will focus on the synergistic mechanisms between influenza subtypes and diverse bacteria, leveraging advanced omics and modeling technologies to enable precise prevention and treatment. Approaches such as combined vaccination and immune-response modulation will be important for better managing co-infections. In addition, differences in specimen types, sampling strategies, and diagnostic methods can significantly impact detection yield. It is crucial to address these sources of variability to minimize misclassification and enhance the reliability of conclusions regarding clinical burden, pathogenic mechanisms, and immune interactions in studies of influenza-bacterial coinfection (Xue et al., 2024; Zhang et al., 2025). Furthermore, future research will necessitate thorough diagnostic stewardship (Elbehiry and Abalkhail, 2025; Yi et al., 2025). Metagenomic and next-generation sequencing have the potential to enhance the detection of pathogens in influenza-associated coinfections; however, mere detection does not suffice to establish a causal relationship (Elbehiry and Abalkhail, 2025). Respiratory specimens with low biomass often harbor oral commensals and opportunistic organisms, with results being affected by the burden of host DNA, background contamination, and the specific thresholds of the analytical platform used (Elbehiry and Abalkhail, 2025; Yi et al., 2025). Standardized pre-analytical workflows, along with quantitative or semiquantitative reporting and interpretation frameworks that incorporate clinical context, host-response signals, and traditional microbiology, are crucial for differentiating between colonization and genuine bacterial pathogenesis in the context of influenza (Elbehiry and Abalkhail, 2025). Examples include the enhanced sensitivity of metagenomic next-generation sequencing for challenging pathogens like *Nocardia* when specific positivity criteria and background subtraction are utilized, as well as 16S-based algorithms that distinguish pathogenic streptococci from oral flora (Kurniawan et al., 2025; Chen et al., 2025). The methodological safeguards in place serve to prevent the misidentification of incidental microbes as causative agents of disease, thereby enhancing the precision of mechanistic inferences in research pertaining to influenza-bacterial coinfection (Elbehiry and Abalkhail, 2025; Kurniawan et al., 2025; Chen et al., 2025). Simultaneously, the advancement of biomarkers is anticipated to enhance early detection and treatment methodologies.

#### 4.2.2 Epidemiological shifts and clinical burden of influenza coinfection during the COVID-19 pandemic

Our analysis indicates that “epidemiological shifts and the clinical burden of influenza co-infection during the COVID-19 pandemic” represents another significant research interest in the field of influenza co-infection. In the early COVID-19 pandemic, widespread non-pharmaceutical interventions like mask-wearing, social distancing, and travel restrictions significantly reduced global seasonal influenza activity and co-infection cases (Pun et al., 2024). As these measures were eased, influenza activity rebounded in several regions, often coinciding with continued SARS-CoV-2 transmission, particularly during the typical influenza season.

Bacterial co-infection in COVID-19 patients occurs at a lower rate than historically observed in influenza, with a prevalence of 7%, thus routine antibiotic use is not advised (Lansbury et al., 2020). Respiratory viral co-infection in COVID-19 patients is about 5.01%, with influenza viruses comprising 1.54% (Krumbein et al., 2023). Among these, 73.6% are due to influenza A and 25.1% to influenza B (Varshney et al., 2023). Although less frequent than single infections, co-infection with influenza and SARS-CoV-2 results in poor outcomes, such as deterioration or death, in 15.7% of cases, posing significant clinical challenges (Varshney et al., 2023).

SARS-CoV-2, as a novel pathogen in the setting of influenza co-infection, can infect pulmonary epithelial cells concurrently with the influenza virus (Zarkoob et al., 2022). This dual infection often leads to more severe pulmonary inflammation and tissue damage, heightening the risk of intensive care units admission and invasive mechanical ventilation (Swets et al., 2022), thus increasing mortality risk (Alosaimi et al., 2021; Yu et al., 2021; Stowe et al., 2021). Reports have also highlighted a heightened risk of long-term complications and adverse outcomes in these patients (Yue et al., 2020). The emergence of SARS-CoV-2 has complicated the immunological response to co-infection. Influenza infection can temporarily suppress SARS-CoV-2 replication (Zarkoob et al., 2022), while prior SARS-CoV-2 infection might inhibit influenza virus entry (Kuriakose and Kanneganti, 2023). For instance, a test-negative case-control study showed that individuals with influenza had a 58% lower risk of testing positive for SARS-CoV-2, suggesting competitive inhibition between the viruses (Stowe et al., 2021). Nonetheless, co-infection with SARS-CoV-2 and influenza can worsen immune dysregulation and provoke cytokine storms, exacerbating clinical outcomes (Bai et al., 2021; Vilas et al., 2022).

Patients with co-infections often exhibit symptoms indistinguishable from those with singular infections of COVID-19 or influenza, complicating clinical diagnosis and treatment (Varshney et al., 2023). This overlap particularly endangers vulnerable populations. The similarity in clinical presentations delays timely diagnosis and appropriate therapeutic decisions, underscoring the need for enhanced biomarker identification and nucleic acid detection methods (Carbonell et al., 2023). To address co-infection risks, robust surveillance of both influenza and SARS-CoV-2 is crucial, alongside ongoing vaccination efforts. Developing vaccines effective against both pathogens could be a cost-effective strategy to alleviate public health burdens and reduce complications from co-infections (Sanchez-Martinez et al., 2024).

#### 4.2.3 Epidemiology and management of influenza-respiratory virus coinfection in pediatric populations

Our bibliometric analysis highlights “epidemiology and management of influenza–respiratory virus co-infection in pediatric populations” as the third prominent research hotspot. Children, due to their developing immune systems and regular exposure to pathogens in environments like daycare facilities and educational institutions, are highly vulnerable to viral co-infections. A study by (Mandelia et al. 2021) revealed that approximately 10.8% of samples from respiratory viral infections exhibited co-infections. Interestingly, the incidence of co-infections was significantly higher in children, accounting for 18% of cases, compared to only 2.8% in adults, indicating a nearly six-fold contrast. Notably, the majority of these co-infections were observed in children under the age of five (Mandelia et al., 2021). Some studies incredibly suggest that as many as 93% of such occurrences happen among children (Weidmann et al., 2023). This underscores the importance of giving priority to children in studies on co-infections linked to influenza. In outbreaks of influenza, around 26.3% of individuals face co-infections, commonly with rhinovirus, adenovirus, or RSV, with children being the most affected group (Torner et al., 2024). These co-infections in children often lead to more serious consequences, such as heightened need for intensive care, prolonged hospitalization, and intricate treatment regimens, emphasizing the significance of pediatric viral co-infection as a notable public health issue (Mandelia et al., 2021).

Our analysis of keyword clusters revealed cluster 2, emphasizing “childre” and “RSV” (Table 6), highlighting crucial research areas. RSV is a primary cause of acute lower respiratory tract infections in children under five, potentially resulting in pneumonia and increasing the likelihood of long-term respiratory problems such as asthma (Pacheco et al., 2021). Despite the significant socio-economic impact of human RSV, there is currently no approved vaccine (Pacheco et al., 2021). A Danish study revealed that influenza cases with various respiratory pathogens, including RSV, are more common in children under 5 years old and decline with increasing age (Schneider et al., 2020). Furthermore, the presence of SARS-CoV-2 presents a notable challenge in cases of pediatric influenza co-infections. In the U.S. during the 2021–2022 influenza season, 6% of pediatric influenza hospitalizations involved co-infections with SARS-CoV-2, and 16% of influenza-related pediatric fatalities were associated with co-infections. These instances often required invasive mechanical ventilation or noninvasive respiratory support such as BiPAP/CPAP (Adams et al., 2022).

Accurate diagnosis and management play a critical role in preventing influenza co-infection among children. Evidence suggests that timely identification of viral infections through point-of-care testing, based on clear clinical indications, can reduce unnecessary antibiotic use and shorten hospital stays (Schneider et al., 2020). This approach is particularly advantageous for children under 5 years old and outside of influenza/RSV seasons (Schneider et al., 2020). Prompt initiation of antiviral treatment is also crucial in preventing co-infections associated with influenza (Xu et al., 2023). Live attenuated vaccines are indispensable for safeguarding vulnerable populations, such as children (Ryan et al., 2022). It is imperative to enhance the research, development, and distribution of vaccines targeting influenza and other respiratory viruses, with a special focus on children (Adams et al., 2022).

### 4.3 Clinical progress

This research synthesizes and critically evaluates six pivotal clinical trials, elucidating a tripartite framework for contemporary clinical investigations on influenza co-infection: targeted protection for vulnerable populations; the nuanced ecological implications of vaccination and its clinical significance; and the imperative to transform future research paradigms.

Initially, in prevention strategies and safeguarding high-risk populations, clinical practice is advancing toward risk stratification to implement precision public health measures that are customized according to varying degrees of vulnerability. In immunosuppressed individuals, such as pediatric patients post-chemotherapy, a multifaceted strategy is essential: enhancing active immunization through vaccination, bolstering passive protection via improved infection prevention and control measures, and implementing early and accurate diagnostic techniques, such as multiplex PCR, to address the dangerous interplay between viral and bacterial pathogens (Tantawy et al., 2015). For healthcare professionals facing elevated exposure risks, empirical evidence underscores the efficacy of N95 respirators over medical masks in mitigating bacterial colonization and co-infection; thus, N95 should be established as the standard for protection in high-risk environments (MacIntyre et al., 2014).

Secondly, concerning the interplay between influenza vaccination and microbial ecology, a cutting-edge subject, our assessment uncovers a complex and dual perspective. The LAIV typically exhibits transient and mild effects on the nasopharyngeal microbiome (Peno et al., 2025); however, under specific circumstances (e.g., co-infection with other viruses), it may temporarily facilitate the proliferation of opportunistic bacteria such as *streptococcus pneumoniae* (Peno et al., 2021). This indicates a phenomenon that possesses both advantageous and disadvantageous aspects. On one hand, baseline microbiome structures may enhance mucosal immune responses (Peno et al., 2025). Conversely, alterations in the local immune microenvironment induced by vaccines may unintentionally establish brief periods that promote bacterial colonization (Hales et al., 2020; Peno et al., 2021). Nevertheless, the existing conclusions are constrained by methodological limitations, such as inadequate resolution of 16S rRNA and qPCR, absence of transmission and disease endpoints, as well as issues related to sample representativeness. The identified tensions suggest a distinct research agenda: the adoption of metagenomics, longitudinal sampling, and the connection to clinical endpoints to clarify mechanisms and quantify absolute risk.

In conclusion, the existing evidence substantiates the need for tailored protection strategies for high-risk groups and calls into question conventional models for assessing the benefit-risk ratio of vaccines. This highlights the necessity for research driven by mechanisms at broader population scales to enhance clinical practice.

### 4.4 Limitations

This study outlines emerging research directions and identifies key hotspots in the field of influenza co-infection, providing valuable insights for future exploration. However, several limitations must be acknowledged. First, our bibliometric analysis primarily utilized the WoSCC database. Although this may have led to the exclusion of some relevant publications, the Web of Science platform is widely recognized for its rigorous curation, quality standards, and reliability in bibliometric studies, offering a robust foundation for our analysis. To address this limitation, we supplemented our assessment with clinical progress data from PubMed-based clinical trials. Second, our analysis was restricted to English-language publications, excluding potentially relevant research in other languages. Third, There exists significant variability in the operational definition of “co-infection” among the primary studies incorporated within our corpus. There are additional variations in laboratory criteria for pathogen confirmation, such as qPCR cycle-threshold cut-offs, microbiological culture interpretation standards, and the kinds of clinical specimens examined (e.g., upper vs. lower respiratory tract samples). The variability in definitions and methodologies leads to the potential for misclassification bias. Consequently, it is imperative to exercise caution when engaging in cross-study comparisons and synthesizing findings. While we have emphasized these issues in the discussion part, they continue to represent a fundamental limitation on the strength and comparability of our conclusions. Lastly, we did not conduct an in-depth author analysis. A significant portion of influenza co-infection research originates from China, where common surnames complicate accurate author disambiguation, potentially affecting author-specific metrics. Despite these limitations, our study provides a comprehensive overview of the current research landscape, effectively mapping prevailing hotspots and emerging trends in influenza co-infection research.

## 5 Conclusion

Our study identifies the main research hotspots and frontiers in influenza co-infection. The key findings are:

A. Influenza co-infection research has garnered significant global interest, with the United States, China, Germany, the United Kingdom, and France as leading contributors. These countries engage in extensive and in-depth collaboration.
B. “PLOS ONE” and “BMC Infectious Diseases” are leading journals in publishing studies in this field, with “Journal of Virology” being the most frequently cited. “Journal of Virology” and “PLOS ONE” likely have a significant impact on influenza co-infection research.
C. The investigation into the pathogenic mechanisms and immune interactions of influenza-bacterial co-infection is a major research focus.
D. The epidemiological shifts and clinical impact of influenza co-infection during the COVID-19 pandemic have gained prominence in recent studies.
E. Research has also concentrated on pediatric populations as a key focus for influenza and respiratory viral co-infections.
F. Clinical trials in this area primarily address the increased risk of severe co-infections in certain groups, such as immunocompromised individuals or those with high-risk genetic profiles, emphasizing the need for targeted prevention. Additionally, studies on the transient effects of influenza vaccination on the respiratory microbiome and bacterial colonization offer valuable insights into co-infection risk.

In conclusion, our study provides valuable insights into the research trends and focal points within the realm of influenza co-infection. These results enhance researchers' understanding and scholarly discourse on this subject. Additionally, our analysis underscores emerging trends and potential avenues for future exploration. By elucidating the present research landscape and pinpointing areas warranting additional scrutiny, this study imparts crucial insights and recommendations for researchers. It aids in informed decision-making and fosters innovation in forthcoming investigations on influenza co-infection.

## Data Availability

The original contributions presented in the study are included in the article/supplementary material, further inquiries can be directed to the corresponding author/s.
