# Supplementary material for: The global trends and clinical progress in influenza co-infection: a visualization and bibliometric analysis (2005–2025)
Source: Front Microbiol. 2025 Oct 8;16:1658752. doi: 10.3389/fmicb.2025.1658752 (PMC12540439; doi:10.3389/fmicb.2025.1658752)
Supplement: Supplementary file 1 [file Data_Sheet_1.docx]

Table S1. Inclusion of Clinical Trials on Influenza Co‑Infections

| Number | Publication year | Title |
| --- | --- | --- |
| 1 | 2014 | Efficacy of face masks and respirators in preventing upper respiratory tract bacterial colonization and co-infection in hospital healthcare workers |
| 2 | 2015 | One-Year Prospective Study of Community Acquired Influenza and Parainfluenza Viral Infections in Hospitalized Egyptian Children with Malignancy: Single Center Experience |
| 3 | 2019 | Evaluation of Mannose Binding Lectin Gene Variants in Pediatric Influenza Virus-Related Critical Illness |
| 4 | 2020 | Symptoms associated with influenza vaccination and experimental human pneumococcal colonisation of the nasopharynx |
| 5 | 2021 | The effect of live attenuated influenza vaccine on pneumococcal colonisation densities among children aged 24-59 months in The Gambia: a phase 4, open label, randomised, controlled trial |
| 6 | 2025 | Interactions between live attenuated influenza vaccine and nasopharyngeal microbiota among children aged 24-59 months in The Gambia: a phase 4, open-label, randomised controlled trial |

Table S2. Exclusion of Clinical Trials on Influenza Co‑Infections

| Number | Publication year | Title |
| --- | --- | --- |
| 1 | 2007 | Effects of pegylated interferon alfa-2b on the pharmacokinetic and pharmacodynamic properties of methadone: a prospective, nonrandomized, crossover study in patients coinfected with hepatitis C and HIV receiving methadone maintenance treatment |
| 2 | 2009 | Improvement of natural resistance in children for prophylaxis of influenza and acute respiratory tract viral infections (results of multicenter randomized trials) |
| 3 | 2010 | Influenza virus contamination of common household surfaces during the 2009 influenza A (H1N1) pandemic in Bangkok, Thailand: implications for contact transmission |
| 4 | 2011 | Vaccination of influenza a virus decreases transmission rates in pigs |
| 5 | 2012 | The role of facemasks and hand hygiene in the prevention of influenza transmission in households: results from a cluster randomised trial; Berlin, Germany, 2009-2011 |
| 6 | 2013 | Aerosol transmission is an important mode of influenza A virus spread |
| 7 | 2014 | Epidemiology of pathogen-specific respiratory infections among three US populations |
| 8 | 2015 | Etiology of community-acquired pneumonia and diagnostic yields of microbiological methods: a 3-year prospective study in Norway |
| 9 | 2016 | Viral load is strongly associated with length of stay in adults hospitalised with viral acute respiratory illness |
| 10 | 2016 | Brief Report: CD14brightCD16- monocytes and sCD14 level negatively associate with CD4-memory T-cell frequency and predict HCV-decline on therapy |
| 11 | 2016 | Streptococcus pyogenes Pneumonia in Adults: Clinical Presentation and Molecular Characterization of Isolates 2006-2015 |
| 12 | 2016 | Viruses as Sole Causative Agents of Severe Acute Respiratory Tract Infections in Children |
| 13 | 2017 | The presence of fever in adults with influenza and other viral respiratory infections |
| 14 | 2018 | Viral etiology of acute respiratory infections in hospitalized children in Novosibirsk City, Russia (2013 - 2017) |
| 15 | 2019 | Maternal Influenza Vaccination and the Risk of Laboratory-Confirmed Influenza Among Household Contacts Under the Age of Five in Mali |
| 16 | 2020 | COVID-19 survival associates with the immunoglobulin response to the SARS-CoV-2 spike receptor binding domain |
| 17 | 2020 | Detection of community-acquired respiratory viruses in allogeneic stem-cell transplant recipients and controls-A prospective cohort study |
| 18 | 2020 | Assessment of indirect protection from maternal influenza immunization among non-vaccinated household family members in a randomized controlled trial in Sarlahi, Nepal |
| 19 | 2020 | Early treatment with baloxavir marboxil in high-risk adolescent and adult outpatients with uncomplicated influenza (CAPSTONE-2): a randomised, placebo-controlled, phase 3 trial |
| 20 | 2022 | Clinical characteristics and illness course based on pathogen among children with respiratory illness presenting to an emergency department |
